# Investigations on the efficacy of routinely used phenotypic methods compared to genotypic approaches for the identification of staphylococcal species isolated from companion animals in Irish veterinary hospitals

**DOI:** 10.1186/2046-0481-66-7

**Published:** 2013-05-01

**Authors:** Lisa Geraghty, Mary Booth, Neil Rowan, Andrew Fogarty

**Affiliations:** 1 Biosciences Research Institute, Athlone Institute of Technology, Dublin Rd., Athlone, Co. Westmeath, Ireland; 2 Department of Life and Physical Science, School of Science, Athlone Institute of Technology, Dublin Rd., Athlone, Ireland

**Keywords:** Companion animals, Staphylococci species identification, Genotyping, tuf, *rpo*B

## Abstract

**Background:**

Identification of Staphylococci to species level in veterinary microbiology is important to inform therapeutic intervention and management. We report on the efficacy of three routinely used commercial phenotypic methods for staphylococcal species identification, namely API Staph 32 (bioMérieux), RapID (Remel) and Staph-Zym (Rosco Diagnostica) compared to genotyping as a reference method to identify 52 staphylococcal clinical isolates (23 coagulase positive; 29 coagulase negative) from companion animals in Irish veterinary hospitals.

**Results:**

Genotyping of a 412 bp fragment of the staphylococcal *tuf* gene and coagulase testing were carried out on all 52 veterinary samples along with 7 reference strains. In addition, genotyping of the staphylococcal *rpo*B gene, as well as PCR-RFLP of the *pta* gene, were performed to definitively identify members of the *Staphylococcus intermedius* group (SIG). The API Staph 32 correctly identified all *S. aureus* isolates (11/11), 83% (10/12) of the SIG species, and 66% (19/29) of the coagulase negative species. RapID and Staph-Zym correctly identified 61% (14/23) and 0% (0/23) respectively of the coagulase-positives, and 10% (3/29) and 3% (1/29) respectively of the coagulase-negative species.

**Conclusions:**

Commercially available phenotypic species identification tests are inadequate for the correct identification of both coagulase negative and coagulase positive staphylococcal species from companion animals. Genotyping using the *tuf* gene sequence is superior to phenotyping for identification of staphylococcal species of animal origin. However, use of PCR-RFLP of *pta* gene or *rpo*B sequencing is recommended as a confirmatory method for discriminating between SIG isolates.

## Background

Staphylococcal species are considered to be opportunistic pathogens, colonising the skin and mucous membranes of humans and animals. In animals, both coagulase positive and coagulase negative Staphylococci have been associated with infection, with a variety of sources identified [1,2]. International animal surveillance programmes on emerging trends in antibiotic resistance typically focus on food animals. Consequently, there is a dearth of similar information for pets or companion animals that frequently are administered with antibiotics, particularly in veterinary hospitals. Recent studies have reported a link between the isolation of multi-drug resistant bacteria from pet owners with companion animal carriage [1]. Antibiotic resistance to beta-lactams, including methicillin resistance, has been found in both coagulase negative and coagulase positive Staphylococci carried by healthy and infected cats, dogs and horses [3-5] reported that various coagulase negative species of Staphylococci from bovine milk differed significantly from each other in antimicrobial resistance profiles (both genotypic and phenotypic) with implications for treatment and management decisions. Accurate speciation of Staphylococci is vital to establish critical links between bacterial species of clinical origin and emerging trends in antibiotic resistance.

Historically, identification of bacterial specimens has been based on conventional microbiological procedures, which include growth on various media, cell morphology, staining reactions and biochemical profiles [6]. Currently in Ireland commercial systems such as API Staph 32 (bioMérieux), Rap-ID (Remel) and Staph-Zym (Rosco Diagnostica) are regularly used to identify species of Staphylococci. There is an absence of published information on the efficacy of using these commercial phenotypic methods for routine Staphylococcal identification particularly from companion animals. Recent reports suggest that phenotypic methods have inherent weaknesses due to the variability in expression of phenotypic characteristics by isolates belonging to the same species and their reliance on subjective interpretation of test results that can also introduce variability [7-11]. Blaiotta and co-workers [8,12] reported a large variation in phenotypic properties of Staphylococci isolated from fermented sausages using laboratory-prepared basal media supplemented separately with fermentable sugars. This group also reported that 25% of these Staphylococci were not identifiable using phenotypic methods. Most phenotypic identification systems have been developed for human healthcare and validated using clinical isolates obtained from human infections [9,11]. When employed on isolates of animal origin, the identification system may be less reliable due to the lack of animal isolates in the reference databases [13,14].

Genotypic methods are reported to have higher discriminatory power, reproducibility and typeability compared to phenotypic methods [10,11,15,16]. Several approaches are available for genotyping bacterial isolates including AFLP (amplified fragment length polymorphism), ribotyping, PCR-RFLP and DNA sequencing. DNA sequence based species identification of Staphylococci is currently the most accurate method with the largest reference database, and is considered to be the gold standard method [10,11]. Traditionally, the most common target for DNA sequencing in bacteria is 16S-rRNA [11,17]. However, this gene is highly conserved among Staphylococci, and often does not provide sufficient discriminatory power to differentiate closely related staphylococcal species [11]. Alternatives to the 16S rRNA gene which have been successfully applied to staphylococcal genotyping include *gap*[17], *cpn* 60 [18], *tuf*[9], *rpo*B [19], *nuc*[20] and *sod*A [21]. Heikens et al., [9] first proposed partial amplification and sequencing of the *tuf* gene as a reliable and reproducible method for the identification of species of Staphylococci. Subsequent studies have confirmed *tuf* gene sequencing as an accurate method for speciating coagulase negative Staphylococci, [22,23]. Blaiotta et al. [24] revealed diversity among coagulase positive *Staphylococcus* species strains based on partial *kat* (catalase) gene sequences and reported a PCR-RFLP assay for identification of coagulase-positive species (*S. aureus, S. delphini, S. hyicus, S. intermedius, S. pseudomedius, S. schleiferi* subsp. *coagulans*). Similarly, Sasaki et al. [25] used a multiplex-PCR method targeting the staphylococcal thermonuclease gene (*nuc*) to successfully differentiate between the same species.

Based upon best-published literature, there is a dearth of critical information on the efficacy of using phenotypic “quick tests” to identify staphylococcal species associated with companion animals. Therefore, this study aimed to compare the efficacy of three routinely-used phenotypic staphylococcal identification test kits with genotypic methods in order to identify the most accurate method of speciating clinical isolates of Staphylococci from a variety of companion animals (dogs, cats, horses) in primary care veterinary hospitals throughout Ireland.

## Methods

### Bacterial strains

Fifty two staphylococcal clinical isolates of veterinary origin and 7 reference control strains were analysed in this study. Clinical specimens were isolated from infection site swabs taken at primary care veterinary clinics (Table 1). Animal species included feline (n=21), canine (n=18), equine (n=11) and bovine (n=2). Infection types included flesh wounds and superficial abscesses (n=38), deep seated infections (n=9), post-operative infections (n=4), and ocular infections (n=3). Swabs were streaked onto Columbia blood agar and Staphylococci were identified by colony morphology, haemolysis patterns, Gram’s stain characteristics, catalase activity, growth on Baird Parker agar, coagulase activity and Voges-Proskauer (VP) testing. Two different coagulase tests were performed: a tube test for free coagulase and a slide test for bound coagulase of clumping factor. Seven control strains and fifty-two clinical isolates were tube coagulase tested according to Murray et al. [6] as follows: a mixture of a 0.1 ml nutrient broth incubated with overnight culture was mixed with 0.5 ml of reconstituted rabbit plasma containing EDTA (Rabbit Coagulase Plasma, Cruinn Diagnostics) in a sterile glass tube and incubated at 37°C in a water bath for 4 hrs. The tubes were observed for clot formation by gently tilting at a 90° angle from the vertical. The tubes were then re-incubated and re-read at 24 hrs. Any degree of clotting was read as a positive result. The slide coagulase test was performed using a Staphylase test kit (Oxoid) according to manufacturer’s instructions. A positive result was read when clumping occurred within 10 seconds. Reference strain identity and source are shown in Table 2.

**Table 1:** Source of Staphylococci isolated from companion animals in veterinary hospitals

**Isolate no.**	**Staphylococcal**	**Companion**	**Infection type**	**Location of veterinary hospital**
	**species**	**animal**		
				**(Region of Ireland)**
1	*S. aureus*	equine	Joint infection	north
2	*S. aureus*	feline	Flesh wound	north
3	*S. aureus*	feline	Face abscess	west
4	*S. aureus*	bovine	Post caesarian	west
5	*S. aureus*	feline	Interdigital infection	west
6	*S. aureus*	feline	Interdigital infection	west
7	*S. aureus*	equine	Abdominal trauma	south
8	*S. aureus*	canine	Post ortho op	east
9	*S. aureus*	feline	Cat fight abscess	west
10	*S. aureus*	feline	Flesh wound	west
11	*S. aureus*	equine	Flesh wound	west
12	*S. pseudintermedius*	canine	Post ortho op	west
13	*S. pseudintermedius*	canine	Granuloma type	north
14	*S. pseudintermedius*	canine	Pyoderma	north
15	*S. pseudintermedius*	canine	Flesh wound	north
16	*S. pseudintermedius*	feline	Deglove wound	west
17	*S. pseudintermedius*	canine	Flesh wound	south
18	*S. pseudintermedius*	canine	Flesh trauma	west
19	*S. pseudintermedius*	equine	Abdominal wound	south
20	*S. pseudintermedius*	equine	Joint infection	north
21	*S. pseudintermedius*	canine	Ear tip infection	west
22	*S. pseudintermedius*	feline	Granuloma type	east
23	*S. pseudintermedius*	canine	Flesh wound	south
24	*S. felis*	canine	Flesh wound	north
25	*S. felis*	feline	Ocular infection	east
26	*S. felis*	canine	Post hysterectomy	west
27	*S. equorum*	canine	Eyelid infection	south
28	*S equorum*	canine	Flesh wound	north
29	*S. equorum*	canine	Face pyoderma	north
30	*S. equorum*	canine	Ear infection	north
31	*S. equorum*	feline	Trauma to face	south
32	*S. equorum*	feline	Cat fight abscess	south
33	*S equorum*	equine	Uterine infection	east
34	*S. equorum*	feline	Flesh wound	south
35	*S. succinus*	equine	Uterine infection	west
36	*S. warneri/pasteuri*	canine	Eyelid infection	south
37	*S. warneri/pasteuri*	bovine	Chronic mastitis	west
38	*S. carnosus/simulans*	feline	Abscess	south
49	*S. carnosus*	feline	Rhinitis	north
40	*S. carnosus*	feline	Pyoderma	north
41	*S. carnosus /simulans*	feline	Puncture wound	west
42	*S. carnosus/simulans*	equine	Chronic flesh wound	west
43	*S. carnosus/simulans*	feline	Abscess	south
44	*S. carnosus*	feline	Ear infection	west
45	*S. carnosus/simulans*	feline	Puncture wound	west
46	*S. carnosus /simulans*	equine	Uterine infection	east
47	*S. xylosus*	feline	Laceration flesh	east
48	*S. xylosus*	canine	Flesh wound	east
49	*S. xylosus*	equine	Uterine infection	west
50	*S. xylosus*	canine	Deglove RTA	south
51	*S. saprophyticus*	feline	Flesh wound	north
52	*S saprophyticus*	feline	Infection in paw	south

**Table 2:** Identification of staphylococcal reference strains

**Species ID**	Source*	***tuf***	***rpo*B**	***pta*-RFLP**	**API Staph 32**	**RapID**	**Staph-Zym**
*S. aureus* IMD247	Athlone Institute of Technology	*S. aureus*	*-*	*-*	*S. aureus*	*S. aureus*	*S. vitulans*
*S. hycius* 11249	University College Dublin	*S. hycius*	*-*	*-*	*S. hycius*	*S. aureus*	*S. hycius*
*S. aureus* 25923	ATCC	*S. aureus*	*-*	*-*	*S. aureus*	*S. aureus*	*No result*
*S. aureus* 43300	ATCC	*S. aureus*	*-*	*-*	*S. aureus*	*S. aureus*	*S. vitulans*
*S. intermedius CCUG* 6520	University of Copenhagen	*S. intermedius*	*S. intermedius*	*S. intermedius*	*S. intermedius*	*S. intermedius*	*No result*
*S. delphini* M4	University of Copenhagen	*S. delphini*	*S. delphini*	*S. delphini*	*S. intermedius*	*No result*	*S. vitulans*
*S. pseudinter -medius* Y19	University of Copenhagen	*S. pseudinter -medius*	*S. pseudinter -medius*	*S. pseudinter -medius*	*S. intermedius*	*S. intermedius*	*S. vitulans*

*American Typed Culture Collection (ATCC).

### Phenotypic identification testing

Phenotypic identification to species level was carried out using three test kits which are commercially available to veterinary laboratories in Ireland, i.e. API Staph 32 (bioMérieux), RapID (Remel) and Staph-Zym (Rosco Diagnostica). Prior to testing, isolates were cultured overnight at 37°C on Columbia blood agar. Tests were carried out according to manufacturers’ instructions and results were interpreted using the appropriate laboratory computer software or reference indices recommended by the manufacturer. The results of the phenotypic tests in this study are also described in terms of sensitivity, specificity and predictive value positive (PVP) of the three test kits and were calculated in comparison with *tuf* genotyping [5]. Sensitivity was calculated as the proportion of the true positive isolates that are correctly identified with the phenotypic tests. Specificity was calculated as the proportion of the true negatives that are correctly identified with the phenotypic tests. The predictive value positive (PVP) for each test was calculated as the proportion of isolates identified as a specific species based on phenotypic testing that truly represented that particular species.

### Genotypic identification testing

Genomic DNA from overnight liquid cultures in nutrient broth was extracted using a DNeasy kit (QIAGEN) according to manufacturer’s instructions. For genotyping, a 412 bp fragment of the *tuf* gene was amplified for all clinical isolates and reference strains according to Heikens et al. [9]. Amplification of a 750 bp *rpoB* gene segment was carried out on all SIG isolates (3 reference strains and 12 clinical isolates) according to Drancourt and Raoult [19], with the following modifications in PCR cycling conditions: 2 minutes at 95°C for 1 cycle, 30 seconds at 94°C, 30 seconds at 47°C, 1 minute at 72°C for 35 cycles, and 5 minutes at 72°C for 1 cycle.

### DNA sequencing

A total of 59 *tuf* amplicons (from 7 reference and 52 clinical isolates) and 15 *rpoB* amplicons (3 reference and 12 clinical isolates) were sequenced by Sequiserve, Germany and Functional Biosciences, USA using amplification primers [9,19]. Forward and reverse sequences were analysed using the BLASTn alignment program and the NCBI nucleotide database NCBI [26].

### PCR-RFLP to differentiate SIG species

Twelve clinical isolates were identified by both *tuf* and *rpo*B genotyping as members of the *Staphylococcus intermedius* group (SIG). PCR-RFLP involving amplification of the *pta* gene followed by digestion with *Alu*1 was used in this study to distinguish between the three known species in the SIG group according to Bannhoer et al., [27] and Kadlec et al. [28].

## Results

### Comparative use of phenotypic ‘quick tests’ and genotypic methods to identify reference strains of staphylococcal species

Seven reference staphylococcal strains were analysed in this study using each of the speciation methods: *tuf* genotyping, API Staph 32, RapID and Staph-Zym. The results are displayed in Table 2. The *tuf* genotyping correctly identified all reference strains. The API Staph 32 test correctly identified *S. aureus*, *S. hycius*, and *S. intermedius* reference strains. However *S. pseudintermedius* and *S. delphini,* were both misidentified indicating that this test does not distinguish SIG species. The RapID test correctly identified all of the *S. aureus* reference strains and *S. intermedius*, but misidentified *S. hycius* and *S. pseudintermedius.* RapID gave no result for *S. delphini*. Staph-Zym identified only one (*S. hycius*) of the 7 control strains, correctly. The three SIG reference strains in the study were also analysed by *rpo*B genotyping and PCR-RFLP of the *pta* gene and were correctly identified by both tests.

### Speciation of staphylococcal clinical isolates

Based on using *tuf* genotyping as the reference identification method for this study, the clinical isolate collection (n=52) was found to comprise of a range of staphylococcal species. The results are shown in Table 3. Forty four percent (23/52) were identified as coagulase positive species, of which 47.8% (11/23) were *S. aureus* and 43% (10/23) were *S. pseudintermedius* (Table 3). For two of the coagulase positive isolates, *tuf* did not distinguish between *S. pseudintermedius* and *S. delphini* (Isolate No’s. 18 and 23). All twelve SIG clinical isolates were confirmed to be *S. pseudintermedius* according to banding patterns using PCR*-*RFLP of the *pta* gene and *rpo*B genotyping. Fifty six percent (29/52) of isolates were identified by *tuf* genotyping as coagulase negative Staphylococci (CONS) (Table 3). These included, *S. equorum* (n=8), *S. xylosus* (n=4), *S. carnosus/simulans* (n=6), *S. carnosus* (n=3), *S. felis* (n=3), *S. warneri*/*pasteuri* (n=2), *S. saprophyticus* (n=2) and *S. succinus* (n=1). For six of the CONS isolates, *tuf* genotyping did not distinguish between two closely related coagulase negative species, identifying them as *S. carnosus* or *S. simulans,* a finding which correlates with research carried out by Ghebremedhin et al. [29]*.* For two additional isolates a result of *S. pasteuri* or *S. warneri* was obtained*.* The results of *tuf* genotyping were consistent with the coagulase test results in all cases with five exceptions, namely *S. aureus* (Isolate No. 1) and *S. pseudintermedius* (Isolate No’s 16, 17, 22 and 23). These strains failed to coagulate plasma in both slide and tube coagulation tests, but were subsequently confirmed as coagulase positive species by PCR-RPLP of the *pta* gene and *rpo*B sequencing.

**Table 3:** Identification of coagulase positive and negative staphylococcal clinical isolates

**No**	***tuf***	**API Staph 32**	**RapID**	**Staph-Zym**
1	*S. aureus*	*S. aureus*	*S. aureus*	no result
2	*S. aureus*	*S. aureus*	*S. aureus*	*S. capitus*
3	*S. aureus*	*S. aureus*	*S. capitus*	no result
4	*S. aureus*	*S. aureus*	*S. aureus*	no result
5	*S. aureus*	*S. aureus*	*S. aureus*	no result
6	*S. aureus*	*S. aureus*	*S. aureus*	no result
7	*S. aureus*	*S. aureus*	*S. aureus*	*S. warneri*
8	*S. aureus*	*S. aureus*	*S. aureus*	*S. warneri*
9	*S. aureus*	*S. aureus*	*S. aureus*	*S. vitulans*
10	*S. aureus st398*	*S. aureus*	*S. aureus*	no result
11	*S. aureus st398*	*S. aureus*	*S. gallinarum/xylosus*	*S. warneri*
12	*S. pseudintermedius*	*S. intermedius*	*S. xylosus*	*no result*
13	*S. pseudintermedius*	*S. intermedius*	*S. gallinarum/xylosus*	*no result*
14	*S. pseudintermedius*	*S. intermedius*	*S. xylosus*	*no result*
15	*S. pseudintermedius*	*S. intermedius*	*S. intermedius/xylosus*	*S. capitus*
16	*S. pseudintermedius*	*S. intermedius*	*S. capitus*	*no result*
17	*S. pseudintermedius*	*S. intermedius*	*S. intermedius*	*S. capitus/hycius*
18	S. pseud/delphini*	*S. intermedius*	*S. gallinarum/xylosus*	*no result*
19	*S. pseudintermedius*	*S. intermedius*	*S. epidermidis*	*S. vitulans*
20	*S. pseudintermedius*	no result	*no result*	*no result*
21	*S. pseudintermedius*	*S. intermedius*	*S. intermedius*	*no result*
22	*S. pseudintermedius*	*S. intermedius*	*S. intermedius*	*no result*
23	S. pseud/delphini*	*Kocuria rosea*	*S. hominus/ capitus*	*S. lentus/fleurettii*
**No**	***tuf***	**API 32**	**RapID**	**Staph-Zym**
24	*S. felis*	*S.chromogens*	*S.chromogenes*	*S. vitulans*
25	*S. felis*	*S. carnosus*	*S. intermedius*	*S. capitus*
26	*S. felis*	*s. xylosus*	*S. xylosus*	no result
27	*S. equorum*	*S. xylosus*	*S. xylosus*	no result
28	*S. equorum*	*S. warnerei*	*S. warnerei*	no result
29	*S. equorum*	no result	*S. xylosus*	*S.xylosus/scuiri*
30	*S. equorum*	*S. xylosus*	*S. xylosus*	no result
31	*S. equorum*	no result	no result	no result
32	*S. equorum*	*S. xylosus*	*S. xylosus*	*S. lentus*
33	*S. equorum*	*S. xylosus*	*S. xylosus*	no result
34	*S. equorum*	*S. epidermidis*	*S. epidermidis*	no result
35	*S. succinus*	*S. xylosus*	*S. xylosus*	*no result*
36	*S.warnerei/ pasteureii*	*S. warnerei*	*no result*	*S. vitulans*
37	*S.warnerei/ pasteureii*	*S. carnosus*	no result	*S.lentus/fleurettii*
38	*S. carnosus /simulans*	*S. carnosus*	no result	*S. warnerei*
39	*S. carnosus*	*S. simluans*	*S. simulans*	*S. carnosus*
40	*S. carnosus*	*S. carnosus*	no result	no result
41	*S. carnosus/simulans*	*S. simulans*	no result	*S.capitus/ hyicus*
42	*S. carnosus/simulans*	*S. simulans*	no result	*S. vitulans*
43	*S. carnosus/simulans*	*s. xylosus*	*S. xylosus*	*S. lentus*
44	*S. carnosus*	no result	no result	no result
45	*S. carnosus/simulans*	*S. xylosus*	*S. xylosus*	no result
46	*S. carnosus/simulans*	*S. equorum*	*S. xylosus*	*S.xylosus/conhii*
47	*S. xylosus*	*S. xylosus*	*S. xylosus*	*no result*
48	*S. xylosus*	*S. xylosus*	*no result*	*no result*
49	*S. xylosus*	*S. intermedius*	*no result*	*S. vitulans*
50	*S. xylosus*	*S. xylosus*	*S. xylosus*	*S. scuiri*
51	*S. sapraphyticus*	*S.sapraphytics*	*S. xylosus*	*S. lentus*
52	*S. sapraphyticus*	*S. xylosus*	*S. xylosus*	*no result*

*= *S. pseudintermedius* or *S. delphini.*

The results of the phenotypic tests in this study are summarised in Table 4, where the sensitivity, specificity and predictive value positive (PVP) of the three test kits are calculated in comparison with *tuf* species identification. The API Staph 32 test showed greatest sensitivity for *S. aureus* isolates (100%). A majority (10/12) of the *S. pseudintermedius* isolates were positively identified as members of the SIG group (83.3%), however, none were accurately speciated. The remaining *S. pseudintermedius* isolates gave either no result (n=1) or was misidentified (n=1). Specificity was 100% for *S. aureus*, and 95% for the SIG group. For the remaining 29 isolates, which were CONS, 31% (9/29) were correctly identified. Isolates identified by *tuf* genotyping as *S. carnosus/simulans* were correctly identified by API Staph 32 as either *S. carnosus* or *S. simulans* in six out of nine cases (66.6% sensitivity). *S. xylosus* species were correctly identified by API Staph 32 with a sensitivity of 75%. In this test, specificity for these two species was 95.3 and 83.3% respectively. Sensitivity was 0% for *S. equorum*, *S. feli*s and *S. succinus* while specificity was 97.7, 98 and 100% respectively. In the case of *S. warneri* and *S. saprophyticus* sensitivity was 50%, with 100% specificity in each case. The API Staph 32 demonstrated the highest PVP with the coagulase positive isolates, with *S. aureus* at 100% and the SIG group at 83.3%. PVP values could not be interpreted for some coagulase negative species, as the test did not identify the species in some cases. *S. xylosus* demonstrated a PVP of 27.2%, due to the high number of false positive results and low specificity (83.3%). Overall, the API Staph 32 had a sensitivity value of 61.5%, a specificity value of 98% and a PVP value of 80% compared to identification of staphylococcal species by *tuf* genotyping.

**Table 4:** Sensitivity, specificity and predictive value positive of API Staph 32, RapID Staph and Staph-Zym tests showing individual results for species isolated in this study and overall values for test kits

**Species**	**API Staph 32**			**RapID**			**Staph-Zym**		
	Sens^1^	Spec^2^	PVP^3^	Sens	Spec	PVP	Sens	Spec	PVP
Overall	61.5	98	80	32.7	95.4	47.2	1.9	99	20
*S. aureus*	100	100	100	81.8	100	100	0	100	n/a
SIG	83.3	95	83.3	41.6	95.2	71.4	0	95	n/a
*S. felis*	0	98	n/a^4^	0	98	n/a	0	98	n/a
*S. equorum*	0	97.7	n/a	0	100	n/a	0	100	n/a
*S. succinus*	0	100	n/a	0	100	n/a	0	100	n/a
*S. warneri*	50	98	50	0	98	n/a	0	98	n/a
*S. carnosus*	66.6	95.3	75	11.1	100	100	11.1	100	100
*S. xylosus*	77	83.3	27.2	50	66.6	15.8	0	66.6	n/a
*S. saprophyticus*	50	100	50	0	100	n/a	0	100	n/a

Values calculated using *tuf* genotyping as a reference method.

^1^Sensitivity ^2^specificity ^3^predictive value positive ^4^not applicable.

RapID correctly identified 81.8% (9/11) of the *S. aureus* isolates (sensitivity 81.8%, specificity 100%), but misidentified all of the *S. pseudintermedius* isolates. On consideration of ability to identify the SIG group, the RapID test had a sensitivity of 41.6% and specificity of 95.2%. Only 7% (2/29) of the CONS were correctly identified, 58.6% (17/29) were misidentified, and no result was obtained for 34% (10/29). When attempting to identify *S. xylosus*, RapID demonstrated a sensitivity value of 50%, but with a specificity of 66.6%, resulting in a PVP of just 15.8%. Overall, the RapID Staph had a sensitivity value of 32.7%, a specificity value of 95.4% and a PVP value of 47.2% compared to identification of staphylococcal species by *tuf* genotyping.

Staph-Zym correctly identified none of the coagulase positive isolates in this study, and only one of the CONS isolates (Isolate No. 39; *S. carnosus*). For 11.5% (6/52) of isolates, Staph-Zym yielded more than one species name. For 44.2% (23/52) Staph-Zym yielded “no result”.

## Discussion

The results presented herein emphasise the importance of choosing the correct identification test for accurate speciation of staphylococcal species of animal origin. The accurate identification of staphylococcal species impacts directly and positively on treatment outcomes and on the epidemiological analysis of emerging trends in multi-drug resistant staphylococcal infections in veterinary medicine.

This present study revealed that all three phenotypic test systems yielded inaccurate speciation results when compared to *tuf* genotyping (Table 3). When considering phenotypic test kits on their own, one must consider the reliability of reading a result with a high “apparent” accuracy. For example, RapID identified one isolate as *S. xylosus* with a 97% probability value; however this isolate was subsequently identified by *tuf* as *S. pseudintermedius*, which could mislead the diagnostician. One of the arguments for using phenotypic test kits is that they are less costly than genotyping. When comparing the costs of phenotyping one must consider the potential consequences of misidentification including unnecessary morbidity and mortality of infected animals.

Of the phenotypic tests utilized, the API Staph 32 correctly identified 100% of *S. aureus* isolates, 83.3% of SIG isolates and 31% of the CONS; the RapID test correctly identified 81.8% of *S. aureus*, 33% of SIG isolates and 6.8% of the CONS; while the Staph-Zym test correctly identified only 2% of all isolates. Each of these tests is based on the evaluation of expression of genetically encoded characteristics by bacterial isolates. Inaccurate speciation may be due to variable expression of biochemical traits within species, as previously reported by Blaiotta et al. [8]. This is supported in the present study where it was observed that in each of the test systems, some biochemical tests frequently gave a misleading response for a given species tested. In particular, tests for arginine dihydrolase, arginine arlyamidase, β-glucuronidase, fructose and mannitol fermentation, novobiocin resistance and nitrate reduction, were observed in one or more systems to generate a response contrary to the expected result for a given species (data not shown). In addition, species identification kits such as these are manufactured for the human diagnostics market and are interpreted against databases with reference strains of human origin. This suggests that the reproducibility and therefore reliability of these tests is questionable when applied to veterinary isolates. When the identification of *S. aureus* by the three phenotyping test kits is considered, it was observed that while all of the *S. aureus* isolates were identified by API Staph 32, they were not consistently identified by either Rap-ID or Staph-Zym, demonstrating a lack of correlation between tests systems for a commonly isolated species. In addition, of the eight *S. equorum* isolates identified by genotyping, API Staph 32 identified four as *S. xylosus*, one as *S. epidermidis,* one as *S. warneri* and did not identify two, suggesting within species variability for the test system. With respect to the newly recognised *Staphylococcus intermedius* group [30], the failure of the phenotypic test kits to correctly speciate members of this group is of concern due to the relevance of *S. pseudintermedius,* not only as a veterinary pathogen, but as a source of nosocomial infection [31].

DNA sequencing of housekeeping genes is regularly used to definitively type staphylococcal isolates, [9,18-21,32]. In this study, *tuf* and *rpoB* gene segments were amplified by PCR and sequenced according to published methods ([9,19], respectively). Both genes constitute more discriminatory targets than the 16S-rRNA gene to differentiate closely related staphylococcal species. The results of this study demonstrated 100% accuracy for reference strains using *tuf* genotyping. Among the clinical isolates, 23 were identified by *tuf* genotyping as coagulase positive species (Table 3). Interestingly, five of these isolates failed to coagulate in both the tube and slide agglutination tests. These findings are not atypical however. According to Murray et al. [6] up to 30% of *S. aureus* field isolates fail to display coagulase activity. Reduced coagulase activity in *S. aureus* is also reported to be associated with reduced susceptibility to vancomycin [33]. It is worth noting that in the present study, each of the four *S. pseudintermedius* isolates which failed to coagulate also showed reduced susceptibility to vancomycin (data not shown). *tuf* genotyping identified 29 coagulase negative isolates and 5 distinct species (Table 3). The species *S. carnosus* and *S. simulans,* however, could not be definitively differentiated from each other by *tuf* genotyping. Likewise, *S. warneri* and *S. pasteuri* were not differentiated by this method. Previous authors have documented a close phylogenetic link between these pairs of species [29,34,35] and the current findings support this.

All three of the reference SIG species were identified correctly by *tuf* genotyping (*S. intermedius, S. delphini, S. pseudintermedius*). Ten of the clinical isolates were identified as *S. pseudintermedius*, but for two additional isolates, *tuf* could not differentiate between *S. pseudintermedius* and *S. delphini*. In an attempt to clarify the identity of these two isolates, both of canine origin, *rpo*B sequencing and PCR*-*RFLP of the *pta* gene were performed. Both isolates were confirmed as *S. pseudintermedius* by the two methods. Given the clinical significance of *S. pseudintermedius* in veterinary medicine, and the published evidence that MRSP (methicillin resistant *S. pseudintermedius*) is emerging as a nosocomial infection, the importance of an accurate identification is paramount. Our findings suggest the use of *rpo*B genotyping or PCR-RFLP of the *pta* gene as a confirmatory method for discriminating between SIG isolates until a larger cohort of these species are entered into the *tuf* gene database, thereby enhancing its accuracy.

## Conclusion

Of the three biochemical tests used, the API Staph 32 test performed with the highest degree of accuracy for the coagulase positive Staphylococci. When compared to *tuf* genotyping all three of the rapid biochemical tests performed poorly for the speciation of coagulase negative Staphylococci. This study highlights the importance of choosing the correct identification test for accurate speciation of staphylococcal species of companion animal origin, as failure to correctly identify specific pathogens may impact on subsequent antimicrobial interventions.

## Competing interests

The authors declare no competing interests.

## Authors’ contributions

LG collected all experimental the data, participated in the study design, sequence alignment and drafting of the manuscript. MB participated in the study design, sequence alignment and drafting of the manuscript. AF participated in the design of the study and drafting the manuscript. NR participated in the study design and drafting of the manuscript. All authors read and approved the final manuscript.
